# Clinical efficacy of magnesium sulfate injection in the treatment of masseter muscle trigger points: a randomized clinical study

**DOI:** 10.1186/s12903-022-02452-3

**Published:** 2022-09-19

**Authors:** Shaimaa Mohsen Refahee, Aliaa Ibrahim Mahrous, Alshaimaa Ahmed Shabaan

**Affiliations:** 1 Oral and Maxillofacial Surgery Department, Faculty of Dentistry, Fayoum University, Fayoum, Egypt; 2 Fixed Prosthodontic Department, Faculty of Dentistry, Fayoum University, Fayoum, Egypt

**Keywords:** Magnesium sulfate, Myofascial trigger point, Masseter muscle, Injection, Saline

## Abstract

**Objective:**

Myofascial pain syndrome with trigger points is the most common cause of nonodontogenic pain. Although injection of the trigger points is the most effective pain reduction treatment, many patients exhibit recurrence after a short period. Therefore, the aim of the current study was to evaluate the clinical efficacy of magnesium sulfate injections in the treatment of the masseter muscle trigger points when compared to saline injections.

**Material and method:**

This study randomly (1:1) assigned 180 patients to one of two treatment groups based on whether their trigger points were injected with 2 ml of saline or magnesium sulfate. Pain scores, maximum mouth opening (MMO), and quality of life were measured at the pre-injection and 1, 3, and 6 months post-injection.

**Results:**

The pain scores were significantly higher in the saline group during all follow-up assessments, whereas the MMO was significantly higher in the magnesium sulfate group up to 3 months of follow-up (*p* < 0.001). However, the difference in MMO ceased to be statistically significant after 6 months of follow-up (*p* = 0.121). Additionally, the patient’s quality of life score was significantly higher in the magnesium sulfate group compared to the saline group (*p* < 0.001).

**Conclusion:**

Injection of magnesium sulfate is an effective treatment measure for myofascial trigger points. However, further studies with a proper design addressing the limitations of the current study are necessary.

*ClinicalTrials*: org (ID: NCT04742140) 5/2/2021.

## Introduction

Myofascial pain syndrome with trigger points (TrPs) is the most common cause of nonodontogenic pain in the orofacial region [1], affecting approximately 40–60% of the adult population [2, 3]. Trigger points are defined as localized areas affected by spasms of the skeletal muscle, inflammation, and low blood flow, typically resulting in localized and referred pain upon palpation, loss of function, sleep disturbances, and a decrease in the patient's quality of life [2, 3].

Muscle pain and fatigue change muscle structure and function. It affects the jaw functions and force by decreasing the firing rate, conduction velocity, and excitability of motor units. In addition to the condylar degeneration which is a bone remodeling response to mechanical pressure [4, 5].

Injection of materials such as local anesthetics, botulinum toxins, corticosteroids, and physiologic saline is an effective method of reducing local and referred pain associated with TrPs. The needling action of these injections and the substances used contribute to successful management of chronic and active trigger points by relaxing the muscle fibers and alleviating pain [6–8]. However, many patients suffer from a recurrence of myofascial pain after a short period of injections, highlighting an unmet clinical need for a new treatment measure that can provide a longer lasting effect [7].

Magnesium sulfate (MgSO4) is commonly used for the treatment of musculoskeletal problems as it has muscle relaxant and vasodilator properties that can have an analgesic effect [9]. These properties can likely be attributed to its ability to block presynaptic acetylcholine discharge from neuromuscular and sympathetic junctions [10]. Moreover, MgSO4 solution can also have an antinociceptive effect in central and visceral pain tests, indicating its potential for use as an adjuvant pain therapy with limited adverse reactions. Previous studies have administered MgSO4 orally or intravenously to reduce pain intensity, particularly in patients with myogenous pain [11]. Yousef et al. [12] found that the use of MgSO4 supplements during the postoperative period in patients with refractory chronic lower back pain reduced pain intensity and improved lumbar spine mobility. The low molecular weight of MgSo4 also makes it suitable for the treatment of deeper tissues due to better penetration, resulting in superior therapeutic effects when compared to injection of other materials used for the treatment of myofascial trigger points [13]. Although previous studies have examined the administration of MgSo4 through oral and intravenous routes, there is limited evidence on the therapeutic effects of injecting MgSO4 into myofascial trigger points. Therefore, the aim of the current study was to investigate the clinical efficacy of MgSO4 injections in the treatment of masseter muscle trigger points when compared to saline injection to evaluate the pain score, maximum mouth opening, and quality of life as clinical parameters.

## Material and methods

This study was conducted between February 2021 and December 2021 at the Oral and Maxillofacial Department, Faculty of Dentistry, Fayoum University. The study was approved by the Beni Suef Research Ethics Committee (code: FDBSUREC/11022021/SA) and the protocol was registered on ClinicalTrials.org (ID: NCT04742140) on 5th February 2021.

**Fig. 1:**
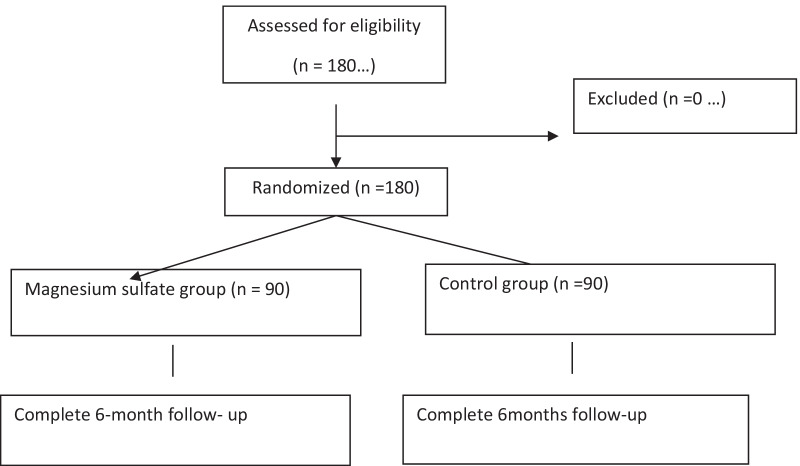
Consort statement flow chart

Informed consent for sharing clinical data and images for scientific purposes was collected from all patients prior to commencement of the study, which was performed in accordance with the Declaration of Helsinki [14] and reported as per the CONSORT guidelines 2012 [15] (Fig. 1).

### Sample size estimation

The primary outcome measure was pain measured using a visual analog scale (VAS). A sample calculation (STATA V16.0) performed using data from a previous study showed that a total of 150 patients would need to be included for a study power of 80% at an alpha of 0.05. However, this study included a total of 180 patients (90 in each group) to account for any possible dropouts [16].

### Study design and randomization

This prospective randomized (1:1) blinded clinical trial used an unstratified random block design (block sizes 2, 4, and 6) to ensure balance in the number of patients assigned to each group.

This study included 180 patients diagnosed with orofacial pain and trigger points in the masseter muscle unilaterally or bilaterally for a period of at least 6 months. The inclusion criteria were as follows: (1) definite diagnosis of myofascial pain based on the DC/TMD criteria with a referral [17]; (2) presence of one or more unilateral or bilateral trigger points in the masseter muscle; and (3) no history of any invasive procedures of the related masseter muscle. The exclusion criteria were (1) any painful conditions (other than myofascial trigger points) affecting the orofacial region; (2) any systemic diseases that could masticatory function (e.g., rheumatoid arthritis and epilepsy); and (3) pregnancy and lactation.

The patients were randomly assigned to one of two groups based on the treatment methods used, as follows: Group I (MgSo4 treatment) and group II (saline treatment).

The primary predictor variable was the treatment method used. The patients, surgeons, and assessors were blinded throughout the period of the study.

### Intervention

After localization of the TrPs, the skin was disinfected with alcohol, the tight muscle band was grabbed between two fingers, and a 30-gage $${\raise0.5ex\hbox{$\scriptstyle 3$} \kern-0.1em/\kern-0.15em \lower0.25ex\hbox{$\scriptstyle 4$}}$$-inch needle was introduced 1–2 cm away from the TrP at a 30º angle to the skin. Negative aspiration was performed and each TrP was injected with 2 ml of either saline or MgSo4 (Magnesium sulfate sterile ampoule 10 ml, 100 mg/ml = 0.41 mMol/ml. Egyptian Int. Pharmaceutical Industries Co., Egypt) [18].

### Outcome measurement

Outcomes, including the pain score, maximum mouth opening (MMO), and quality of life, were measured preinjection and 1, 3, and 6 months after injection.

Pain intensity was measured using a 10-point VAS, where a score of 0 indicated no pain and a score of 10 indicated maximum pain [19]. The MMO was assessed by measuring the interincisal distance between the upper and lower central incisors [19]. The quality of life was assessed using the Oral Health Impact Profile questionnaire (OHIP-14) containing 14 questions divided into seven domains of oral health. The participant was asked to score each question using a scale from 1 to 5 [never (score 0); hardly ever (score 1); occasionally (score 2); fairly often (score 3); and very often (score 4)], and the sum of all 14 items was calculated. The OHIP score could range from 0 to 56 units, with the former indicating no problems and higher scores representing greater impairment of quality of life [20].

### Statistical analysis

Categorical data were presented as frequencies and percentages and were compared using Fisher’s exact test, whereas numerical data were reported as mean and standard deviation values. The Shapiro–Wilk test was used to assess normality, and parametric analyses included independent t-tests for intergroup comparisons and repeated measures analysis of variance and posthoc Bonferroni corrections for intragroup comparisons. Nonparametric analyses included Mann–Whitney U tests for intergroup comparisons and Friedman’s test followed by pairwise comparisons using multiple Wilcoxon signed rank tests with Bonferroni correction for intragroup comparisons. The significance level was set at *p* < 0.05, and all statistical analyses were performed using R, version 4.1.1 for Windows [21].

## Results

This study included 180 cases divided equally into two groups [MgSo4 group n = 90, 15 males and 75 females; saline group n = 90, 13 males and 77 females]. The mean age of the MgSo4 group was 35.91 years, whereas that of the saline group was 30.53 years **(**Table 1).

**Table 1:** Summary statistics of demographic data

Parameter			Magnesium sulphate group	Saline group	*p* value
Gender	Male	n	15	13	0.681
		%	16.7%	14.4%	
	Female	n	75	77	
		%	83.3%	85.6%	
Age	Mean ± SD	35.91 ± 12.61	30.53 ± 8.51	0.001*	

*Significant (*p* < 0.05)

No complications were observed except redness and mild discomfort at the injection site that was seen to resolve within 24 h in both groups.

The pain scores were significantly higher in the saline group compared to the MgSo4 group at all follow-up timepoints (*p* < 0.05; Table 2), whereas the MMO value was significantly higher in the MgSo4 group up to 3 months of follow-up (*p* < 0.001). However, the differences in MMO were no longer statistically significant after 6 months of follow-up (*p* = 0.121; Table 3). The OHIP-14 score was significantly higher in the MgSo4 group compared to the saline group throughout the study period (*p* < 0.001; Table 4).

**Table 2:** Comparison between primary predictable variables and pain score

Parameter	Time	Mean ± SD (95%CI)		*p* value
		Mg sulfate group	Saline group	
Pain	Baseline	7.12 ± 0.99 (6.92–7.33)	7.18 ± 0.77(7.02–7.34)	0.778
	1 month	0.40 ± 0.60 (0.28–0.52)	0.57 ± 0.54 (0.45–0.68)	0.019*
	3 months	0.44 ± 0.60 (0.32–0.57)	2.36 ± 0.68 (2.22–2.50)	< 0.001*
	6 months	1.94 ± 0.77 (1.79–2.10)	4.48 ± 0.90 (4.29–4.66)	< 0.001*

*Significant (*p* < 0.05)

**Table 3:** comparison between primary predictable variables and maximum mouth opening

Parameter	Time	Mean ± SD (95%CI)		*p* value
		Mg sulfate Group	Saline Group	
Maximum mouth opening (mm)	Baseline	30.55 ± 1.43 (30.26–30.85)	31.34 ± 1.17 (31.10–31.58)	< 0.001*
	1 month	34.36 ± 0.95 (34.17–34.56)	33.92 ± 0.66 (33.79–34.06)	< 0.001*
	3 months	34.33 ± 0.96 (34.13–34.53)	32.72 ± 0.66 (32.59–32.86)	< 0.001*
	6 months	32.77 ± 1.04 (32.56–32.99)	32.50 ± 1.16 (32.26–32.74)	0.092

*Significant (*p* < 0.05)

**Table 4:** comparison between primary predictable variables and quality of life score

Parameter	Time	Mean ± SD (95% CI)		*P* value
		Mg sulfate Group	Saline Group	
Quality of life	Baseline	35.23 ± 4.21 (34.36–36.10)	40.44 ± 3.89 (39.64–41.25)	< 0.001*
	1 month	1.78 ± 0.95 (1.58–1.97)	0.62 ± 0.92 (0.43–0.81)	< 0.001*
	3 months	13.68 ± 4.31 (12.79–14.57)	3.64 ± 2.72 (3.08–4.21)	< 0.001*
	6 months	24.62 ± 2.95 (24.01–25.23)	13.98 ± 2.57 (13.45–14.51)	< 0.001*

*Significant (*p* < 0.05)

## Discussion

Myofascial pain syndrome is a neuromuscular problem characterized by muscle spasms, pain, and the presence of myofascial TrPs that present as muscle band contractions [17, 22–24]. TrPs most commonly affect the masseter muscles in the orofacial region, and are one of the major triggers of nonodontogenic pain [25, 26].

Because myofascial muscles are a part of the stomatognathic system, imbalance in any part of this system could have a detrimental impact on its function such as chewing, posture and non-physiological occlusion.that affect the patient's quality of life [27].

Injection of myofascial TrPs with different materials such as saline, local anesthesia, botulinum toxin, and platelet rich plasma injections can help reduce pain and has been seen to be widely tolerated [22, 28, 29]. However, many patients exhibit recurrence of myofascial pain after a short period of injections, highlighting the unmet clinical need for a new treatment measure with a longer lasting effect.

The aim of the current study was to evaluate the clinical efficacy of MgSo4 injections in the treatment of masseter muscles with TrPs when compared to saline injections. MgSo4 has been recommended for the treatment of myofascial TrPs due to its muscle relaxant and vasodilator properties that can have a pain-relieving effect. Few studies till date have examined the effects of MgSo4 on various musculoskeletal inflammatory disorders [9–12].

This study included 180 patients, and 84.4% of both study groups were female. This findings, in agreement with previous epidemiological evidence on tempero-mandibular disorders, can be attributed to hormonal and bio-behavioral factors, a higher demand for treatment among females, and their increased tendency toward psychological disorders [30, 31].

The mean age of patients in the MgSo4 group was 35.91 ± 12.61 years whereas that in the saline group was 30.53 ± 8.51 years, and this was in agreement with previous evidence that found that myofascial TrPs typically occurred in patients aged between 27 and 50 years [32].

The current study used the VAS scale to estimate pain intensity at each study interval, and significantly lower values were observed in patients receiving MgSo4 injections at all follow-up intervals when compared to patients receiving saline injections (*P* < 0.05). This could likely be attributed to the increased vasodilation provided by the former in several vascular beds, resulting in greater blood flow to the trigger point and removal of irritating substances that cause pain. Additionally, it also eliminates muscle tension and excessive tenderness by competing with calcium at the motor end plate and reducing acetyl choline discharge [33, 34]. This, in turn, leads to reduction of pain intensity at the site of injection, and these findings are in harmony with those of Ibrahim et al. [9], who also reported observing a palliative effect following iontophoresis with MgSO4 in healthy adult volunteers. Furthermore, Sane et al. [35], studied the effect of local injection of ropivacaine and bupivacaine injection with magnesium sulfate on postoperative pain in vertebral laminectomy surgery and concluded that local anesthesia combined with magnesium sulfate provided greater postoperative analgesia and considerably reduced postoperative opioid use.

On the other hand, Ahmed et al. [36], tested the efficacy of ultrasound-guided erector spinae plane block with and without the addition of magnesium sulphate on pain control in patients with postherpetic neuralgia and found that the addition of magnesium sulphate made no difference when compared to the use of bupivacaine alone.

The MMO was significantly higher in the MgSO4 group up to 3 months of follow-up (*p* < 0.001), although this statistical significance ceased to exist after 6 months (*p* = 0.121). Increased tension in the muscular band, inhibition of motor activity, muscle shortening, and occurrence of spasms often results in hypomobility, and the improvement in MMO observed in this study could be attributed to the muscle relaxation effect of MgSO4 which lowers acetylcholine release at the myoneural junction, blocking peripheral neuromuscular transmissions and inhibiting skeletal muscle contractions [34].

This is harmonized with the finding of Fathy et al. [37], who compared the efficacy of transforaminal MgSO4 injection against Ozone on pain intensity and functional disability in patients with lumbar disc prolapse and concluded that MgSO4 offered better analgesia, noticeably reduced analgesic ingestion and improved the functional disability.

Quality of life is an important parameter when evaluating the outcomes of various treatment measures for chronic pain. OHIP-14 is a specific tool used to assess oral function and measure oral health related quality of life. In the current study, all treatment groups exhibited improvement in the OHIP-14 score at the follow-up assessments, with the MgSo4 injection group exhibiting significantly better outcomes compared to the saline injection group (*P* < 0.001). This could be attributed to a reduction in pain intensity and improvement of function in the former [19, 38–41].

Compromised quality of life has been often reported for patients suffering from chronic pain which recovered with different treatment that improves the pain level [42]. This is in line with Azi et al. [43], who evaluate the analgesic effect of trigger point acupuncture combined with cyclobenzaprine chlorhydrate and sodium dipyrone. Azi et al. concluded that pain relief and improvement in quality of life at 4 weeks in both groups. Moreover, Brodsky et al. [44], evaluated change in health-related quality of life at the group and individual levels in a consecutive series of patients with chronic myofascial neck pain and concluded that considerable improvement over time was found for all scores post-treatment of myofascial neck pain.

The present study had several limitations. First, it did not compare the effects of MgSo4 injections to any other materials such as local anesthesia, botulinum toxin, or platelet rich plasma. Second, objective methods of assessment such as EMG could not be used due to limited resources.

## Conclusion

The findings of this study suggest that MgSo4 injections are an effective treatment modality for myofascial TrPs of the masseter muscle. It reduces the pain and improves the maximum mouth opening, in addition to the quality of life. However, further studies with an improved design that addresses the limitations of the current study are necessary.

## Data Availability

The datasets used and/analyzed in the current study are available upon reasonable request from the corresponding author.

## Abbreviations

TrPs: Trigger points
MgSo4: Magnesium sulfate
VAS: Visual analog scale
MMO: Maximum mouth opening
OHIP-14: Quality of life assessed using oral health impact profile questionnaire
